# Association between HLA-B*1502 allele and aromatic antiepileptic drugs-induced hypersensitivity syndrome reactions and the HLA-B*15:02 pharmacogenetics screening in autistic spectrum disorder

**DOI:** 10.1186/2045-7022-4-S3-P124

**Published:** 2014-07-18

**Authors:** Chonlaphat Sukasem, Nattawat Ngamsamut, Ananya Sinrachatanant, Bhunnada Chamkrachchangpada, Tan-kam Teerarat, Montri Chamnanphon, Apichaya Puangpetch, Penkhae Limsila

**Affiliations:** 1 Laboratory for Pharmacogenomics, Ranathibodi Hospital Mahidol University, Thailand; 2 Yuwaprasart Waithayopathum-Child and Adolescent Psychiatric Hospital, Ministry of Public, Department of Mental Health Services, Thailand; 3 Yuwaprasart Waithayopathum-Child and Adolescent Psychiatric Hospital, Ministry of Public, Department of Mental Health Services, Thailand; 4 Laboratory for Pharmacogenomics, Ranathibodi Hospital Mahidol University, Department of Pathology, Thailand

## Poster presentation

Some autistic children also have symptoms of depression, anxiety or obsessive-compulsive disorder (OCD). Aromatic anti-epileptic drugs (AEDs) may be used as a mood-stabilizing medication to treat these symptoms, including carbamazepine (CBZ), oxcarbazepine (OX-CBZ), and lamotrigene (LMG). Moreover, one in every four autistic spectrum disorder children have seizures, the use of AED becomes imperative in managing symptoms. Previous studies found a strong association between HLA-B*1502 and carbamazepine (CBZ)-induced cutaneous adverse reactions in Thai epileptic patients. This study aimed to identify whether HLA-B*1502 is associated with AEDs-induced hypersensitivity syndrome (HSS) reactions and also determine the frequency of HLA-B*15:02 in autistic spectrum disorder (ASD) in Thailand. In this study, patients that developed fever and cutaneous eruptions in the presence or absence of organ involvement with exposure to carbamazepine (CBZ), phenytoin (PHY), or lamotrigine (LTG) were enrolled. HLA-B* 15:02 was then determined in 21 patients with AED-induced HSS (6 with CBZ-HSS, 4 PHY-HSS patients and 11 LTG-HSS patients and 292 children with ASD. HLA-B genotyping using PCR-SSOP methods was performed. HLA-B*15:02 was observed with HSS-induced by AEDs, which included carbamazepine (n=4; 66.7%), phenytoin (n=2; 50%), and lamotrigine (n=3; 27%). In addition, the allele frequencies of HLA-B*15:02 in ASD was 14.72% (43/292). HSS-induced by CBZ, PHY and LMG is strongly, moderately and slightly associated with HLA-B*15:02 in Thai patients, respectively. Our findings suggest that HLA-B*15:02 testing before AED therapy would be effective at risk of hypersensitivity and applicable to ASD populations providing hope for prevention in the future.

